# tANCHOR fast and cost-effective cell-based immunization approach with focus on the receptor-binding domain of SARS-CoV-2

**DOI:** 10.1093/biomethods/bpad030

**Published:** 2023-12-12

**Authors:** Hubert Bernauer, Anja Schlör, Josef Maier, Norbert Bannert, Katja Hanack, Daniel Ivanusic

**Affiliations:** ATG:biosynthetics GmbH, Merzhausen 79249, Germany; new/era/mabs GmbH, Potsdam 14482, Germany; Institute for Biology and Biochemistry, University of Potsdam, Potsdam 14476, Germany; ATG:biosynthetics GmbH, Merzhausen 79249, Germany; Robert Koch Institute, Berlin 13353, Germany; new/era/mabs GmbH, Potsdam 14482, Germany; Institute for Biology and Biochemistry, University of Potsdam, Potsdam 14476, Germany; Robert Koch Institute, Berlin 13353, Germany

**Keywords:** tANCHOR, immunization, GPCR, SARS-CoV-2, CD20, antibodies

## Abstract

Successful induction of antibodies in model organisms like mice depends strongly on antigen design and delivery. New antigen designs for immunization are helpful for developing future therapeutic monoclonal antibodies (mAbs). One of the gold standards to induce antibodies in mice is to express and purify the antigen for vaccination. This is especially time-consuming when mAbs are needed rapidly. We closed this gap and used the display technology tetraspanin anchor to develop a reliable immunization technique without the need to purify the antigen. This technique is able to speed up the immunization step enormously and we have demonstrated that we were able to induce antibodies against different proteins with a focus on the receptor-binding domain of SARS-CoV-2 and the extracellular loop of canine cluster of differentiation 20 displayed on the surface of human cells.

## Introduction

Hybridoma technology produces monoclonal antibodies (mAbs) in hybrid cells generated from the fusion of an antibody-secreting B-cell with an immortalized myeloma cell line [1]. In particular, for the generation of mAbs by B-cell hybridoma technology, mice are mainly immunized with a specific antigen [2–5]. In the case of mAb development with recombinant protein antigens, the production can be very time-consuming. It can take several weeks before the antigen is available for immunization. Many antigens are produced in intracellular compartments, which require isolation by cellular lysis, purification by chromatography, and often solubilization or refolding in order to obtain the desired protein conformation [6–9]. Reduction of process steps for antigen production will reduce overall the costs of manufacturing mAbs [10]. In addition, cell-surface membrane proteins may lose their native conformation during purification steps [11]. This may make it challenging to develop specific mAbs against the desired target. A solution is to overexpress the antigen in human embryonic kidney 293T (HEK293T) cells and then immunize the host with these cells [12]. The main advantage of using transfected HEK293T cells is that the antigen is expressed and displayed to the immune system in a natively folded form including all posttranslational modifications. Mammalian expression systems are used when a natively glycosylated protein conformation is desired [13]. Glycosylation of proteins is very critical as shown for HIV when neutralizing antibodies should be raised against native viral protein structures [14]. To meet the requirements for a fast and efficient immunization approach including posttranslational modification of the antigen, we employed the tetraspanin (Tspan) anchor (tANCHOR) system [15, 16]. This system is based on the use of transmembrane domains derived from the Tspan superfamily where a protein of interest is fused and expressed as a chimeric membrane-bound protein displayed on the surface of human cells. Notably, transmembrane anchors derived from the Tspan CD82 show the best performance for displaying proteins or peptides on the cell surface of HEK293T or HeLa cells [15]. The antigen is displayed on the cell surface and presented to the immune system in a native conformation.

We report here the results of the immunization of mice by using HEK293T cells expressing tANCHORed antigen on the surface. As proof of principle, we have focused our immunization strategy on the receptor-binding domain (RBD) of SARS-CoV-2 and the extracellular loop (ECL) of canine cluster of differentiation 20 (cCD20). In particular, the immunization of mice for the investigation of RBD antigenicity enables comparison among different variants. This point is important because the reduced antigenicity of distinct SARS-CoV-2 variants of concern has increased the risk of immune escape variants spreading globally and resulting in pandemic waves [17].

## Material and methods

### Molecular cloning and plasmid DNA preparation

Expression constructs that anchor the antigen on the surface of HEK293T cells were based on the tANCHOR display system described previously [15]. In detail, the commercially available vector ptANCHOR-CD82-V5-His-mCherry (ATG:biosynthetics GmbH, Merzhausen, Germany) was used to insert DNA fragments coding for the SARS-CoV-2 RBD variants (Supplementary Table S1) between EcoRI and EcoRV restriction sites. We used the GISAID database for RBD variant sequence information [18]. DNA fragments and the vector ptANCHOR-CD82-V5-His-mCherry were digested with enzymes EcoRI-HF and EcoRV-HF (NEB, New England Biolabs, Frankfurt, Germany), and restricted DNA fragments were ligated into the restricted vector by using T4 ligase (NEB). The ligation mixture was transformed into chemically competent *Escherichia coli* DH5α (NEB). Inserted DNA sequences between EcoRI and EcoRV were obtained by gene synthesis and code for SARS-CoV-2 RBD amino acids 318–543 (GenBank: YP_009724390.1) Wuhan-Hu-1, Delta B.1.617.2 and Omicron BA.1, Wuhan-Hu-1 RBD amino acids 462–510 (GenBank: YP_009724390.1) and canine CD20 amino acids 162–191 (GenBank: NP_001041493.1). The DNA insert coding for the human angiotensin-converting enzyme 2 (ACE2) protein comprising the amino acids 1–740 fused to a V5-tag (GKPIPNPLLGLDST) [19] and 6xhistidine tag [20] (ACE2_1–740_-V5-His) was cloned using the restriction sites NheI and PmeI and the vector FlexMam-Puro (ATG:biosynthetics). RBD sequences were used as found in the viral genomes, except for the removal of the EcoRI restriction recognition site (GAATTC was replaced by GAACTC). The part of the ACE2 UTR sequence shown in Supplementary Table S1 was originally taken from expression cassettes of the Flex vector series FlexMAM (ATG:biosynthetics GmbH). The CDS of ACE2 was codon-optimized for expression in HEK293 cell lines and purchased from ATG:biosynthetics GmbH. Sequences were confirmed by restriction analysis and Sanger sequencing. The vector pmCherry-N1 expressing monomeric mCherry protein was purchased from Clontech (Heidelberg, Germany). Plasmid DNA for transfection of HeLa and HEK293T cells was prepared from confirmed clones using a Maxiprep plasmid purification kit according to the manufacturer’s instructions (Qiagen, Hilden, Germany). DNA sequences used for molecular cloning are listed in the Supplementary Table S1.

### Cell culture and cell transfection

HEK293T or HeLa cells were maintained in Dulbecco’s modified Eagle’s medium supplemented with 10% fetal bovine serum (Gibco, Thermo Fisher, Germany), 100 IU/ml penicillin, 100 µg/ml streptomycin (Gibco) and 2 mM L-glutamine (Gibco) at 37°C, 5% CO_2_ and relative humidity of about 95%. To prepare cells for immunization, 5 × 10^5^ HEK293T cells were seeded in a 6-well plate (TPP, Techno Plastic Products AG, Trasadingen, Switzerland) and transfected at a confluency of 80% with 5 µg of plasmid DNA and 10 µl of Metafectene transfection reagent according to the manufacturer’s instructions (Biontex Laboratories, Munich, Germany). The transfection mix was added to the cells and incubated for 48 h. The medium was removed and cells from one 6-well plate were resuspended with sterile 1× phosphate-buffered saline (PBS) and collected in one reaction tube. Cells were stored at −80°C before injection into mice.

### Immunization of mice

Six- to 8-week-old BALB/c and C57BL mice were intraperitoneally immunized at each immunization timepoint with 1 × 10^6^ transfected HEK293T cells in 200 µl of PBS. Mice were immunized each week for a period of 4 weeks. After 5 weeks, serum was obtained and tested for antigen-specific antibodies.

### Measurement of antigen-specific serum IgG by protein/peptide-based ELISAs

Total serum content of immunoglobulins G (IgGs) was assessed by enzyme-linked immune sorbent assay (ELISA). Our performed ELISA is designed to detect specific antibody–antigen interactions. The antigen (RBD protein) is immobilized on a solid surface (96-well plate) and bound antibodies derived from mice serum are then detected with an anti-mouse detector antibody that is conjugated to horseradish peroxidase (HRP) as a reporter enzyme. Briefly, ELISA plates (Nunc MaxiSorp, Thermo Fisher) were coated with 100 µl of 1 µM peptide diluted in carbonate buffer (15 mM Na_2_CO_3_, 35 mM NaHCO_3_, pH 9.6) overnight at 4°C. Plates were washed three times with 300 μl of wash buffer PBS-T (1× PBS containing 0.05% Tween-20) and blocked for 2 h with 200 μl of blocking buffer containing 2% chicken egg albumin (Sigma Aldrich, Germany) and 3% bovine serum albumin (Carl Roth) in 1× PBS. We used peptides for screening of IgGs against Wuhan RBD amino acids 462–510 (KPFERDISTEIYQAGSTPCNGVEGFNCYFPLQSYGFQPTNGVGYQPYRVDED; the amino acids DED were added C-terminally to improve the water solubility of the synthesized peptide) and for CD20 amino acids 162–191 (VDIHNCDPANPSEKNSLSIQYCGSIRSVF). Peptides were synthesized by ProteoGenix (Schiltigheim, France). The FLAG immunization control ELISA was performed by coating the ELISA plate with the peptide MDYKDHDGDYKDHDIDYKDDDDK (Intavis Peptide Services, Tübingen, Germany). In order to screen for full-length RBD-specific IgGs, Nunc MaxiSorp plates were coated with 50 µl of 3 µg/ml Wuhan, Delta, and Omicron RBD protein (ProteoGenix) diluted in carbonate buffer. Plates were sealed with an ELISA polyester seal film (Carl Roth, Karlsruhe, Germany) and incubated overnight at 4°C. After coating plates, they were washed with BioTec 405 ELISA washer (Agilent Technologies, Waldbronn, Germany) three times with 300 μl of PBS-T. Serum obtained from immunization was diluted in blocking buffer and incubated for 1 h on coated ELISA plates. Plates were then washed three times with 300 μl of PBS-T wash buffer and bound antibodies were detected by a 45 min incubation step using rabbit anti-mouse-IgG-HRP secondary antibody (P0260, 1.3 mg/ml, Dako, Agilent Technologies, Denmark) at a dilution of 1:10,000, followed by five successive washes with 300 μl of wash buffer. Bound secondary HRP-conjugated antibodies were detected by the addition of 80 μl of TMB (3,3′,5,5′-tetramethylbenzidine) substrate (BioRad, Munich, Germany) and incubated at room temperature for 15–30 min. The reaction was stopped by the addition of 100 μl of 2 M H_2_SO_4_ (Carl Roth). Absorbance values were measured at 450 nm with correction at 620 nm employing an Infinite microplate reader (Tecan, Männedorf, Switzerland). To test immunogenicity against the spike protein, we used the coated plates from the SARS-CoV-2 IgG ELISA kit (EUROIMMUN, Lübeck, Germany) and used it in the same way as described for self-coated plates. The control antibody mouse anti-FLAG-M2-HRP (A8592, 1 mg/ml, Sigma Aldrich, Steinheim, Germany) was used at a dilution of 1:2,000 for detecting coated FLAG-antigen.

### Generation of supernatant containing ACE2_1–__740_-V5-His protein

HEK293T cells were transfected in a 6-well plate at a confluency of 80% with 4 µg of pACE2-V5-His plasmid DNA and 8 µl of Metafectene according to the manufacturer’s transfection instructions. After 24 h, the medium was replaced by a medium containing 5 µg/ml puromycin (Carl Roth). Cells were treated after 3 days with a medium containing 2 µg/ml puromycin. Cells were split with trypsin (Sigma Aldrich) after reaching a dense cell layer; 2 ml of fresh medium was added to a confluent 6-well plate and the supernatant was collected after 3 days. Collected supernatants were stored at −80°C until usage for the binding assay.

### Cell-based ACE2–RBD interaction assay

To examine the ACE2_1–740_-V5-His binding to the RBD variants, we performed a cell-based ELISA as previously described [15]. In detail, we transfected HeLa cells with generated tANCHOR vectors containing coding sequences for the Wuhan, Delta, and Omicron RBDs. The binding of the ACE2_1–740_-V5-His to the RBD was assayed by incubation of 100 µl of the supernatant from cells stably expressing ACE2_1–740_-V5-His protein. Bound ACE2_1–740_-V5-His was detected with mouse anti-V5-HRP (46-0708, 1.18 mg/ml, Invitrogen) at a dilution of 1:8,000 in blocking buffer supplemented with 2% chicken egg albumin (Sigma Aldrich, Germany) and 3% bovine serum albumin (Carl Roth). The TMB reaction was performed as described in the ELISA method section above.

### Western blot analysis

Expressed proteins were detected by Western blot analysis using whole cell lysates for analyzing expressed tANCHORed antigens [21, 22]. Transfected HEK293T cells were lysed for analysis of whole-cell protein expression (6-well plate) by adding 100 µl of 2× Laemmli buffer containing β-mercaptoethanol (Sigma Aldrich, Germany) supplemented with 200 U/ml Benzonase nuclease (Merck/Millipore, Darmstadt, Germany) and 1 µl of 100× Halt protease inhibitor cocktail (Thermo Fisher). After degradation of chromosomal DNA, cell extracts were boiled for 3 min at 90°C. An aliquot of 10 µl of cell extracts was separated with sodium dodecyl sulfate–polyacrylamide gel electrophoresis (SDS–PAGE) by using 4%–15% Protean TGX gels (Bio-Rad, Munich, Germany) and 1× SDS–PAGE buffer for electrophoresis; 10× SDS–PAGE buffer contains 144.4 g of glycine (Carl Roth, Karlsruhe, Germany), 30.3 g of Tris (Carl Roth) and 10 g of SDS pellets (Carl Roth) dissolved in 1 l of water. Separated proteins were transferred to a 0.2 µm PVDF Mini trans-blot membrane (Bio-Rad) and then blocked for 1 h with 10% blotting grade low-fat milk powder (Carl Roth) diluted in pure water. Proteins were detected using the specific primary antibodies 1:2,000 mouse anti-mCherry (TA180028, 1 mg/ml, OriGene, Herford, Germany). The primary antibody was detected by incubation of diluted rabbit anti-mouse-IgG-HRP (P0260, 1.3 mg/ml, Dako) at a dilution of 1:5,000 for 45 min. Human ACE2-specific rabbit antibodies (MA5-32307, 1 mg/ml, Invitrogen, Fisher Scientific, Schwerte, Germany) were used at a dilution of 1:2,000 for 1 h and detected by goat anti-rabbit-IgG-HRP (P0448, 0.25 g/ml, Dako) at a dilution of 1:5,000 for 45 min. The HRP activity was detected by incubation of PVDF membranes with Pierce Super Signal West Pico substrate (Thermo Fischer) and signals were imaged by using a Chemocam digital image analyzer (Intas, Göttingen, Germany).

### Analysis of transfection efficiency by flow cytometry

Transfected cells used for immunization and untransfected (control) HEK293T were pelleted and resuspended in 500 µl of 2% paraformaldehyde (PFA) for 30 min. Cells were then centrifuged and resuspended in 1× PBS. Cells were stored at 4°C until analysis by fluorescence-activated cell-sorting (FACS) analysis by using the red fluorescence protein mCherry reporter protein to detect transfected cells. For this, cells were centrifuged and resuspended in FACS buffer (PBS, 0.01% NaN_3_, 0.5% BSA). Cells were analyzed using a green laser line (Ex 561 nm; filter 610/20) to detect mCherry fluorescence on a FACS Aria III (BD Biosciences, Heidelberg, Germany) to confirm successful transfection. Data were analyzed with FlowJo™ software [23].

### Quantification of mCherry-tagged antigens

Antigens for immunization were quantified by using a mCherry quantification kit (Abcam, Cambridge, UK). Cell pellets of transfected HEK293T and untransfected HEK293T cells (control) were lysed in 1 ml of mCherry assay buffer and incubated on ice for 15 min. Samples were centrifuged to remove cell debris and 100 µl of supernatants was transferred to a 96-well optical plate (Greiner Bio-One, Frickenhausen, Germany). In addition, mCherry standard protein provided with the mCherry quantification kit was used to generate a standard curve (diluted in mCherry assay buffer to obtain 0, 20, 40, 60, 80, 100, 120, 140, 160, 180, and 200 ng/well) from the fluorescence readings to calculate the mean amount of mCherry protein that was extracted from the cell pellet. All fluorescence values were measured using a GloMax-Multi+ detection system in combination with a green filter kit for red dyes (Promega, Walldorf, Germany). The relative fluorescence unit (RFU) background value (RFU for wells containing only mCherry assay buffer) was subtracted from all readings. The amount of antigen that was used for immunization was calculated by Equation (1):


(1) $$m\left(\mathrm{Antigen}\right)=M\left(\mathrm{Antigen}\right)\cdot \frac{m(\mathrm{mCherry})}{M(\mathrm{mChery})}$$

where *m* is the mass of the antigen, *M* is the molar mass of the antigen (RBD_318–543_ Wuhan 25,506.86 g/mol, RBD_318–543_ Delta 25,576.96 g/mol, RBD_318–543_ Omicron 25,796.38 g/mol, RBD_462–510_ Wuhan 5,527.10 g/mol, and cCD20_162–191_ 3,194.54 g/mol), *m (mCherry)* is the mass of quantified mCherry protein and *M (mCherry)* is the molar mass of the fluorescent protein mCherry (26,722.19 g/mol).

### Analysis of protein localization and ACE2_1–740_-V5-His binding to the displayed RBD by cLSM

HEK293T cells (1 × 10^4^/well) were seeded into high glass-bottomed 8-well ibidi μ-slides (ibidi, Munich, Germany) and after 24 h were transiently transfected with 0.5 µg of plasmid DNA and 1 µl of Metafectene transfection reagent. At 24 h post-transfection, cells were washed once with 1× PBS and fixed with 200 μl of 2% PFA in 1× PBS for 15 min. Cells were then washed once with 1× PBS and left in 200 μl of 1× PBS in the presence of 1 μl of Hoechst 33342 (ImmunoChemistry Technologies, CA, USA). To examine the binding of ACE2_1–740_-V5-His to the RBD displayed on the surface, 1 × 10^4^ HeLa cells per well were seeded into high glass-bottomed 8-well ibidi μ-slides and after 24 h cells were transfected with 0.5 μg of plasmid DNA. After 48 h post-transfection, cells were washed once with 1× PBS, fixed for 15 min with PFA, washed with 1× PBS three times, and incubated with 200 μl of supernatant containing ACE2_1–740_-V5-His protein for 1 h. Cells were washed after incubation three times with 1× PBS and incubated with 200 μl of rabbit anti-V5 (NB600-381, 1 mg/ml, Novus Biologicals, Littleton, USA) diluted 1:1,000 in blocking buffer for 1 h, followed by three washing steps with 1× PBS. About 300 μl of goat anti-rabbit-IgG Alexa 488 (A111008, 2 mg/ml, Invitrogen, Fisher Scientific, Schwerte, Germany) diluted 1:2,000 in blocking buffer was finally added for 45 min. All images were acquired using an inverted confocal laser scanning microscope (AxioObserver LSM 780; Carl Zeiss Microscopy GmbH, Oberkochen, Germany) and a plan-apochromat oil immersion objective (63×, numerical aperture 1.4; Carl Zeiss Microscopy GmbH). In this microscopy technique, laser scanning means the images are acquired point by point under defined laser excitation. Furthermore, images are obtained by the focal plane only, because the pinhole removes all emissions not originating from the focal plane and illumination and detection are therefore focused on the same spot [24, 25]. Fluorescence signals were detected with the Zeiss ZEN smart setup single-track settings for the dyes Hoechst 33342 excitation (Ex.) 405 nm/emission (Em.) 464 nm (429–499 nm), 0.2% laser power, Alexa 488 Ex. 488 nm/Em. 546 nm (493–589 nm), 1.5% laser power and mCherry Ex. 594 nm/Em. 648 nm (499–696 nm), 6.9% laser power. Images were scanned unidirectional with a fixed pixel size of 0.13 μm (1024 × 1024 pixels per image).

### Analysis of SARS-CoV-2 neutralization activity

The SARS-CoV-2 neutralization activity of antibodies was assayed utilizing a NeutraLISA system (EUROIMMUN, Lübeck, Germany) according to the manufacturer’s instructions. Serum samples were diluted 1:20 in sample buffer containing biotinylated ACE2. Inhibition values (%) were calculated from absorbance values at 450 nm and reference wavelength 620 nm measured with an Infinite 200 microplate reader (Tecan, Crailsheim, Germany). Cutoff values are defined by the manufacturers as follows: <20%: negative; between ≥20% and <35%: borderline; ≥35%: positive.

### Statistical analysis

Statistical analysis was performed by using GraphPad Prism software version 9.2.0. The unpaired two-tailed Student’s *t*-test was used to analyze the statistical significance comparing the two groups. A *P*-value <.05 was considered as statistically significant, non-significant (ns): >.05, *P < .05, **P <* *.01, ***P <* *.001, ****P <* *.0001. Data are presented as mean* *±* *standard deviation (SD). Data were tested prior to the Student’s *t*-test by the Shapiro–Wilk test for confirming Gaussian distribution.

### Digital illustrations

Illustrations and images were created with BioRender.com, Microsoft Office 2016, and Graph Pad Prism version 9.2.0.

## Results

### Antigen design for cell-based immunization

**Figure 1.**
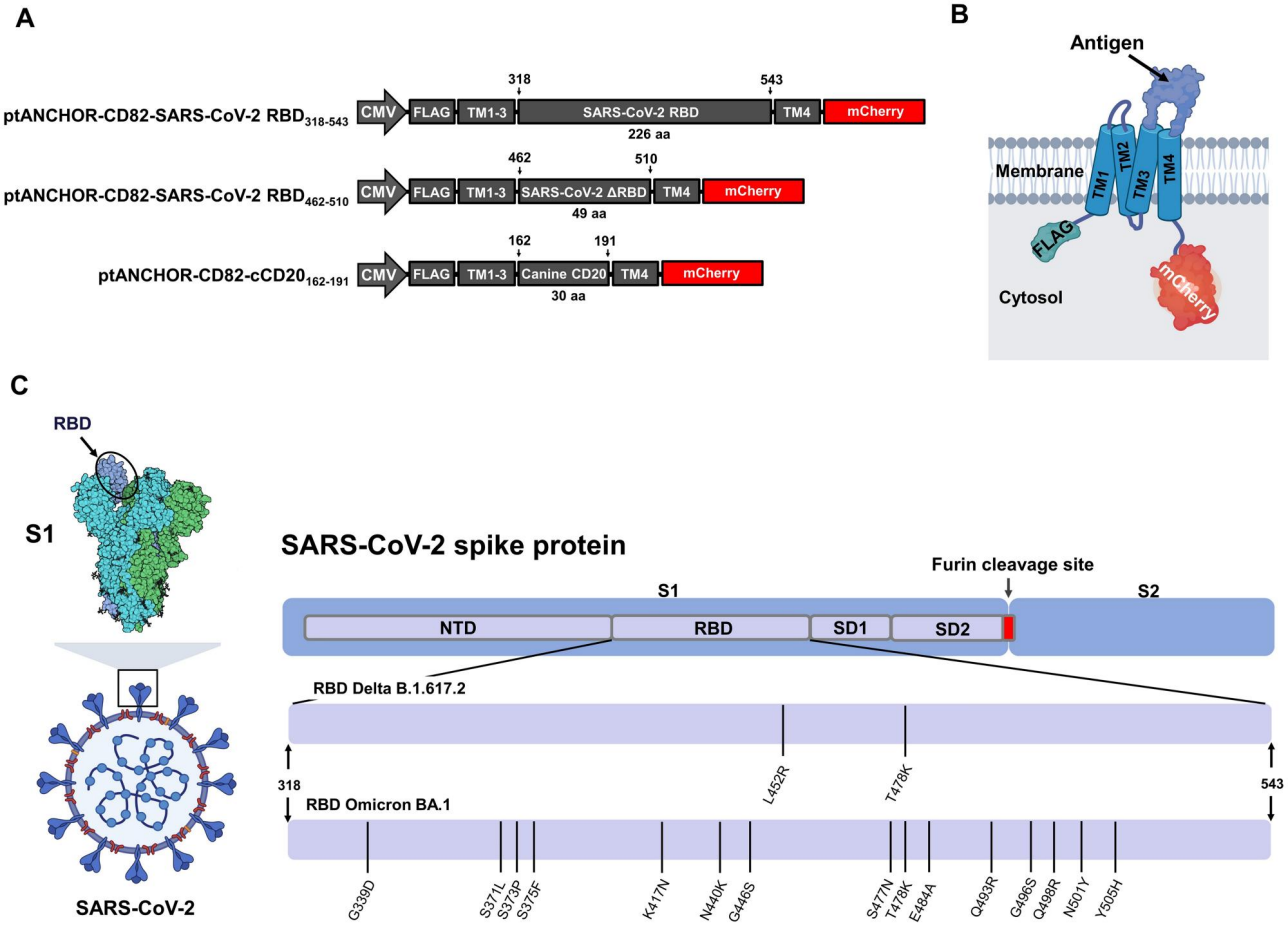
Antigen design for the immunization study. (A) Schematic representation (not to scale) of expression constructs containing the indicated DNA coding for antigens derived from the SARS-CoV-2 RBD and canine CD20 (cCD20). DNA fragments are cloned in the tANCHOR display expression vector backbone (ptANCHOR-CD82). Expression is driven by the cytomegalovirus (CMV) promoter. The constructs encode CD82-derived domains (CD82 without the LEL) for anchoring the antigen on the cell surface between transmembrane domain (TM) 3 and 4 (B) Cartoon showing the topology of extracellular displayed antigen expressed as a chimeric membrane-bound protein. The expressed protein contains an N-terminally FLAG-tag (DYKDDDDK) and C-terminally a monomeric red fluorescent protein (mCherry) as a reporter for protein expression and distribution analysis. (C) Cartoon of the RBD within the spike protein domain 1 (S1) localized on the surface of viral particles (left side). Overview of the introduced mutation sites within the Wuhan-Hu-1 RBD that were used for immunization with Delta and Omicron SARS-CoV-2 variants (right side). N-terminal domain (NTD), spike protein subdomains (SD1 and SD2). Numbers refer to amino acid (aa) positions.

We used the tANCHOR vector ptANCHOR-CD82-V5-His-mCherry to clone the DNA inserts coding for the RBD of SARS-CoV-2 ancestral Wuhan strain and for the canine CD20 between the restriction sites EcoRI and EcoRV (Fig. 1A). The tANCHOR system utilizes a tANCHOR derived from the Tspan CD82. It was generated by deleting the large ECL (LEL) of CD82 in order to fuse the protein of interest between the transmembrane domains 3 and 4. The expressed protein is a chimeric membrane-bound protein where the inserted sequence is exposed extracellularly (Fig. 1B). The C-terminally fused monomeric red fluorescent protein (mCherry) is useful as a reporter protein for expression analysis and monitoring of protein localization [26]. The three main constructs (Fig. 1A) are of different sequence lengths, between 30 and 226 amino acids, to test if shorter or longer antigen designs can be used for the induction of antibodies. In addition, we cloned the Delta (B1.6.17.2) and Omicron (BA.1) RBD variants in the tANCHOR vector system to test the antigenicity of the presented RBDs. The RBD is part of the spike protein (Fig. 1C) and is important for the interaction with the host cell receptor ACE2. The spike protein is expressed as a polyprotein and cleaved by furin protease into subunits S1 and S2. Many of the SARS-CoV-2 neutralizing antibodies target the RBD within the S1 subunit [27–29].

### Expression and characterization of tANCHORed antigen constructs

**Figure 2.**
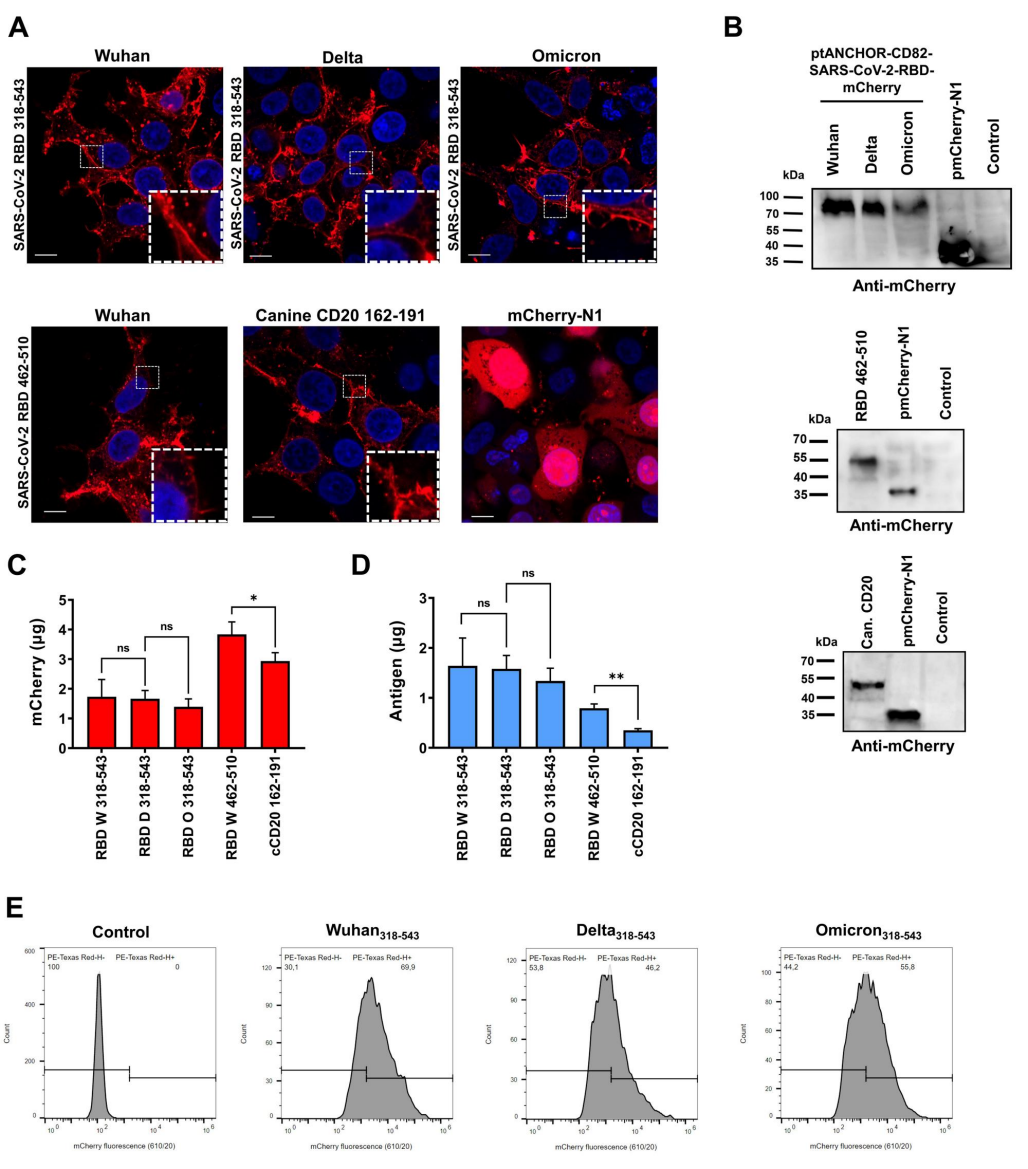
Antigen expression analysis and characterization. (A) cLSM images of HEK293T cells transfected with tANCHOR constructs containing coding DNA sequence for the SARS-CoV-2 RBD or cCD20. As a control for mCherry expression, the cells were transfected with the vector pmCherry-N1; all scale bars 10 µm. (B) Western blot analysis of HEK293T whole cell lysates (10 µl per lane) transfected with tANCHOR vectors containing coding DNA sequences of the Wuhan, Delta, Omicron RBD_318–543_, Wuhan RBD_462–510_ and canine CD20 (cCD20_162–191_). In the case of the Wuhan RBD_462–510_ and cCD20 blots, the mCherry lysate was diluted 1:4 in 2× Laemmli buffer. (C) Quantification of the antigen fusion proteins expressed in HEK293T cells by using the mCherry reporter protein. (D) Calculated antigen amount that was used for immunization. Absorbance values were measured in triplicate. (E) Analysis of transfection efficiency by FACS using mCherry as reporter protein. The height of the bar represents the mean value and the error bar indicate SD, non-significant (ns): *P* > .05, *P < .05, **P < .01. Wuhan (W), Delta (D), and Omicron (O).

To follow up protein localization of expressed tANCHORed antigens fused with the fluorescent protein mCherry, we transfected HEK293T cells and analyzed cellular distribution by confocal laser scanning microscopy (cLSM). We confirmed the cellular distribution as expected on the cell surface in HEK293T cells of all expressed proteins containing different antigens (Fig. 2A). Western blot analysis of lysates from transfected HEK293T cells with indicated plasmids confirmed the expression of variant RBD_318–543_ fused with tANCHOR elements with a molecular mass of ∼85 kDa (Fig. 2B). For the shorter Wuhan RBD_462–510_ sequence, we observed a band at ∼55 kDa and for cCD20 at ∼50 kDa. Next, we quantified the expressed tANCHORed antigens employing mCherry as a fluorescent reporter protein (Fig. 2C). We used the results from the mCherry quantification and calculated the amount of antigen in the cell pellets that were injected into mice during weekly repeated immunizations (Fig. 2D). The cell pellets contained between 1.3 and 1.6 µg of antigen for the RBD with 226 amino acids and 0.8 µg for the shorter RBD with 49 amino acids. For the shortest antigen sequence of cCD20_162–192_, we calculated an antigen amount of 0.4 µg. To compare the transfection efficiency between Wuhan Hu-1, Delta 1.617.2, and Omicron BA.1 RBD_318–543_, we analyzed the cells by FACS and reached a transfection efficiency of 69.9% (Wuhan), 46.2% (Delta), and 55.8% (Omicron) (Fig. 2E). In conclusion, all generated plasmid constructs were correctly expressed and displayed the antigen on the cell surface.

### Proof of native RBD_318–543_ conformation by detection of ACE2 interaction

**Figure 3.**
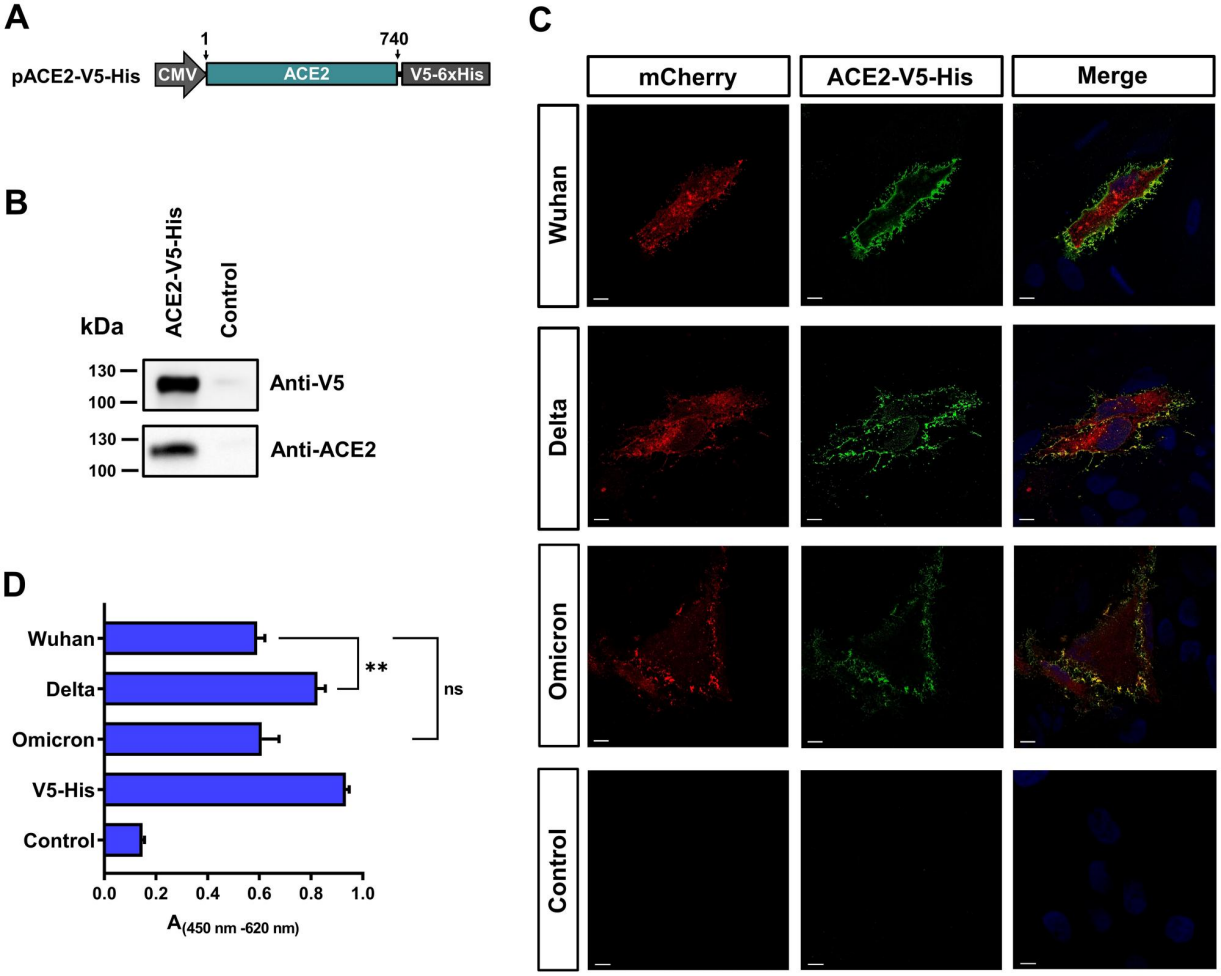
Analysis of the ACE2–RBD interaction. (A) Schematic presentation (not to scale) of the construct used for secretion of the human ACE2 protein fused with a V5 (GKPIPNPLLGLDST) and a 6×histidine tag (ACE2_1–740_-V5-His). The numbers refer to the amino acids. (B) Western blot analysis of the ACE2_1–740_-V5-His protein secreted from HEK293T cells. (C) Analysis of ACE2_1–740_-V5-His protein binding on the cell surface by cLSM of transfected HeLa cells with indicated vectors expressing tANCHORed RBDs. Control was performed in parallel with HeLa cells that were not transfected, scale bars 10 µm. (D) Results of a cell-based ELISA used to analyze binding of secreted ACE2_1–740_-V5-His protein to the expressed RBD on the surface of HeLa cells. Control contains HeLa cells without plasmid transfection. The V5-His antibody control was not incubated with soluble ACE2_1–740_-V5-His protein. Absorbance difference values at 450 nm with baseline correction at 620 nm were measured in triplicate, non-significant (ns): *P* > .05, **P < .01. The height of the bar represents the mean value, and the error bar represent the spread of the values (SD).

Native conformation of expressed proteins is essential to generate antibodies that are reactive against a native protein structure [30, 31]. In the case of a recombinant RBD, the binding affinity to ACE2 reflects a native conformation [32]. We therefore confirmed the binding of RBD_318–543_ to human ACE2 by a HeLa cell-based ELISA approach. This interaction assay was carried out with soluble ACE2 protein. For this, we cloned the fragment coding for the ACE2 amino acids 1–740 in the FlexMam-Puro vector (Fig. 3A). HEK293T cells transfected with the resulting plasmid will secrete truncated ACE2 protein fused with a V5-His detection tag into the culture medium. Successful secretion was confirmed by Western blot analysis (Fig. 3B). Antibodies raised against human ACE2 are reactive against protein ACE2_1–740_-V5-His secreted from stably expressing HEK293T cells (Fig. 3B). Transfected HeLa cells with tANCHOR constructs were used to visualize bound ACE2_1–740_-V5-His protein on the cell surface by an indirect immunostaining technique. All expressed tANCHORed RBDs (amino acids 318–543) were able to bind the secreted ACE2_1–740_-V5-His protein on the cell surface (Fig. 3C). Further, we employed a cell-based ELISA assay to determine the binding efficiency of ACE2_1–740_-V5-His on the cell surface of HeLa cells expressing the indicated RBD. The results showed that there is a difference between the Wuhan and Delta variants. The Omicron RBD binds ACE2_1–740_-V5-His in the same manner as the Wuhan RBD (Fig. 3D) and this result is consistent with a former report [33]. The specific binding of ACE2_1–740_-V5-His with the presented RBD variants confirmed the native conformation of the RBD on the cell surface.

### Immunization and analysis of induced IgG levels

**Figure 4.**
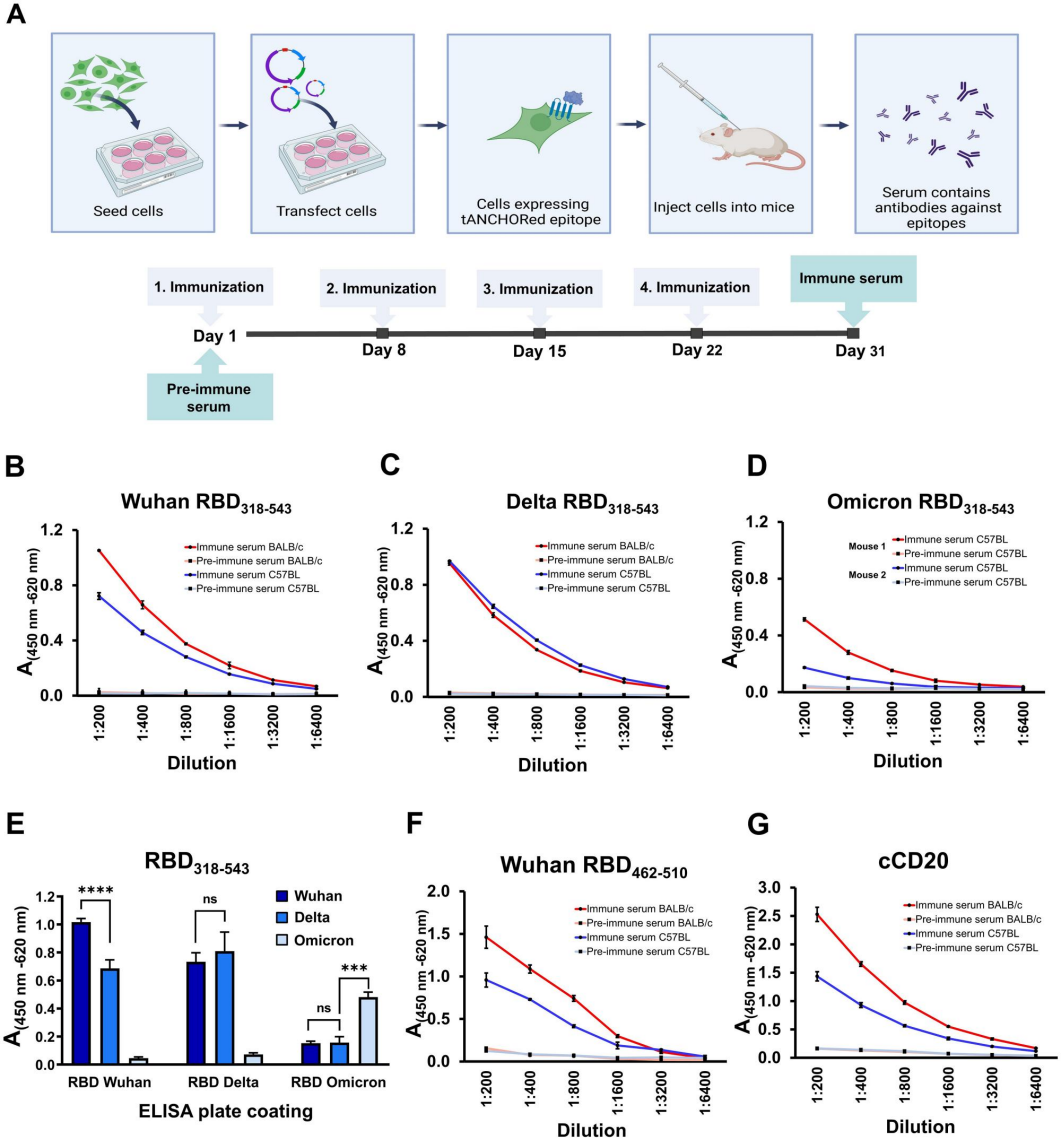
Analysis of serum from immunization by ELISA. (A) Schematic presentation of the cell-based immunization steps performed. The timeline shows the immunization and sampling schedule for serum by arrows. (B–D) Binding of IgG against coated recombinant Wuhan RBD (B), Delta RBD (C) and Omicron RBD (D). (E) Analysis of IgG binding derived from C57BL mice serum bound to different RBDs coated onto plates by ELISA. (F) Analysis of IgG bound to the corresponding peptide derived from the SARS-CoV-2 RBD, amino acids 462–510. (G) Serum from mice immunized with tANCHORed cCD20 was tested by a peptide corresponding to the cCD20 sequence 162–191. Absorbance values were measured in triplicate (B–D, F, and G) or quadruplicate (E). The height of the bar represents the mean value, and the error bars represent the spread of the values (SD), non-significant (ns): *P* > .05, ***P <* *.001, ****P <* *.0001.

HEK293T cells were transfected and resuspended after 48 h from a 6-well plate with PBS. The cells were injected intraperitoneally four times weekly into 6-week-old mice (Fig. 4A). The induction of polyclonal antibodies in BALB/c and C57BL mice against the antigen was evaluated by ELISA. First, we coated the ELISA plate with recombinant RBD protein and tested serum from mice that had been immunized with the full-length RBD. Second, we coated the plates with peptides and tested serum from the mice immunized with the truncated Wuhan RBD (amino acids 462–510) and cCD20. The immune response to displayed antigens in mice was assessed by monitoring of induced specific IgG. The measurement of specific IgG against RBD was chosen because it represents a powerful tool to predict the efficacy of SARS-CoV-2 vaccination [34]. We observed that mice immunized with Wuhan RBD_318–543_ and Delta RBD_318–543_ were able to produce (IgG) antibodies at the same titer but for the Omicron RBD_318–543_ the titer was lower (Fig. 4B–D). The observed variation in antibody titers between two C57BL mice (mouse 1 and 2, Fig. 4D) immunized with the Omicron RBD is also seen in other immunization studies utilizing the RBD as immunogen [32, 35]. Interestingly, the BALB/c strain mice failed to produce reliable IgG levels against the Omicron RBD_318–543_ (Supplementary Fig. S1A and B). Next, we tested variant-specific antibody binding to recombinant RBD proteins. We observed that antibodies induced against the Wuhan and Delta RBD_318–543_ variants showed a difference in binding characteristics. Antibodies generated against the Wuhan RBD showed a higher binding frequency to the Wuhan RBD compared to antibodies raised against the Delta variant. However, when these antibodies were tested on a Delta RBD recombinant protein, we observed that there was no significant difference in their binding, regardless of whether they were raised against the Wuhan or Delta variants (Fig. 4E). In contrast, antibodies against the Omicron RBD showed a lower binding frequency on Wuhan and Delta RBD protein but a higher binding frequency on the Omicron RBD protein (Fig. 4E). These results demonstrate that the performed immunization is suitable to develop specific humoral immune responses against a variant-specific RBD. Even with shorter sequences, it was possible to induce specific antibody responses. For both mouse strains, BALB/c and C57BL, we were able to develop antibodies against a truncated Wuhan RBD version (Fig. 4F) and the 30 amino acid-long sequence derived from canine CD20 (Fig. 4G).

### Analysis of induced IgG level on SARS-CoV-2 spike S1 protein and on a FLAG control peptide

**Figure 5.**
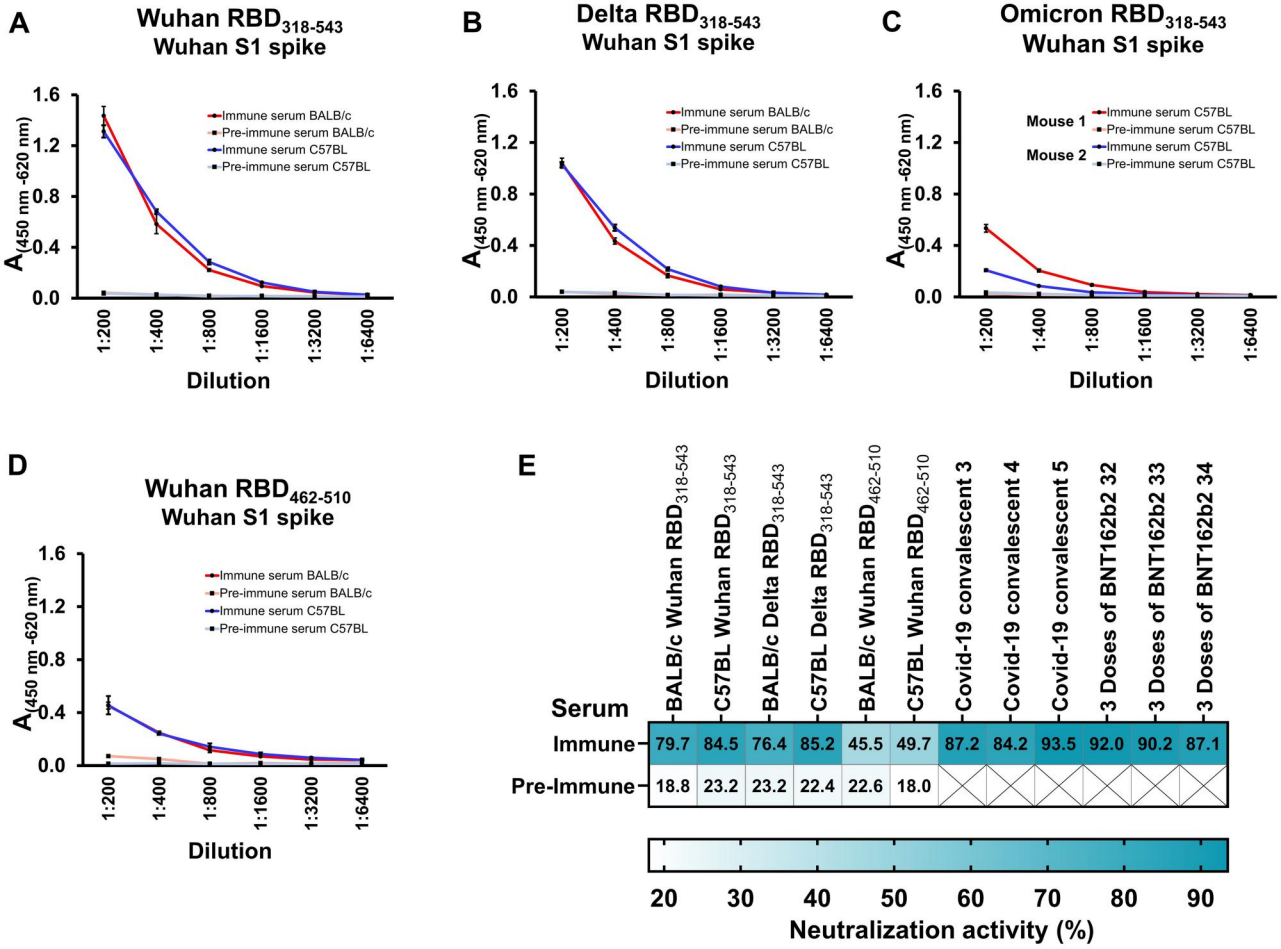
Analysis of immunogenicity against spike protein S1 and neutralization activity. Binding of IgG against S1 protein from immunization with (A) Wuhan RBD_318–543_, (B) Delta RBD_318–543_, (C) Omicron RBD_318–543_ and (D) Wuhan RBD_462–510_. Absorbance values were measured in triplicate, the error bars represent the spread of the values (SD). (E) Analysis of neutralization activity of immune and pre-immune sera, as well as a human control serum at a dilution of 1:20.

We further analyzed the binding of induced IgG antibodies to the SARS-CoV-2 spike subdomain 1 (S1). Serum from mice immunized with Wuhan RBD_318–543_ (Fig. 5A) displayed the highest reactivity against the S1 protein, followed by that from mice immunized with Delta RBD_318–543_ (Fig. 5B). In contrast, mice immunized with the Omicron RBD_318–543_ developed antibodies that were bound nearly three times less efficiently to the spike S1 compared with the ancestral RBD (Fig. 5C). Induced antibodies against the truncated RBD sequence were also able to bind the spike protein. We observed a lower absorbance value that could have resulted from limited binding sites within the whole spike protein (Fig. 5D). All tANCHORed antigens used for immunization were expressed containing an N-terminally fused FLAG-tag [36]. We, therefore, tested induced antibodies against the FLAG-epitope DYKDDDDK and detected only weak binding to the coated FLAG-peptide (Supplementary Fig. S1B). This suggests that the tag that is intracellularly exposed will not be efficiently presented to the immune system. We have already confirmed the intracellular topology of the FLAG-tag in our previous work [15].

### Analysis of neutralization activity by utilizing the NeutraLISA system

Finally, we analyzed pre- and immune serum obtained from the immunization study for neutralization activity. For this, we utilized the surrogate NeutraLISA system. This system is based on the blockage of protein–protein interaction between ACE2 and the Wuhan RBD that is commercially available for testing of neutralizing antibodies (nAb) and strongly correlates with the gold standard method plaque reduction neutralization tests using replication-competent SARS-CoV-2 particles [37, 38]. Because this system contains the Wuhan RBD as an interaction partner for soluble ACE2, we therefore only measured serum obtained from mice immunized using Wuhan and Delta RBD content. It has been shown that infection with either Wuhan (ancestral) or the Delta SARS-CoV-2 variant induces cross-neutralizing antibodies with comparable titers against both viruses [39]. Our results show that the obtained immune serum is able to inhibit the interaction between ACE2 and the RBDs by 79.7%–85.2% (Fig. 5E). We also measured serum derived from COVID-19 (Coronavirus disease 2019) convalescent individuals and from vaccinees who had received three doses of the mRNA vaccine BNT162b2 and observed that mouse serum contains comparable neutralizing activity to that of the COVID-19 convalescent group. Only the group of vaccinated individuals showed higher neutralization activity values. Interestingly, serum from immunized mice with the truncated RBD showed weak neutralization activity of 45.5% and 49.7%. This weaker neutralization is explained by the fact that the identified binding sites for ACE2 between K417 and F456 are missing in the RBD antigen design that was used for immunization [40].

## Discussion

**Figure 6.**
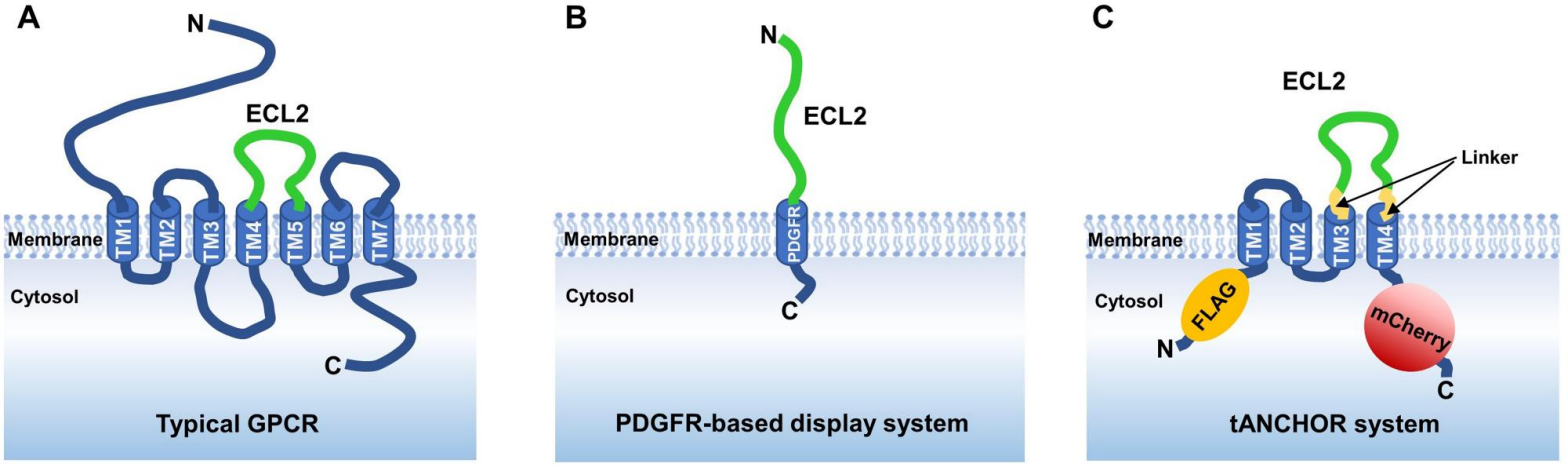
Future perspective of improvements for antigen design targeting ECLs derived from G protein-coupled receptors (GPCRs). (A) Typical topology of a GPCR. TM: transmembrane domain of the GPCR, ECL2: second extracellular loop, N: N-terminus, C: C-terminus. (B) ECL2 displayed by the use of a single membrane domain derived from the PDGFR. (C) Utilizing the tANCHOR system for displaying the ECL2 offers a loop structured antigen design. ECL2 is connected by short linker sequences between the third and the fourth transmembrane domain (TM) derived from CD82. The red fluorescent reporter protein (mCherry) is fused C-terminally and the FLAG-tag N-terminally.

In this study, we developed a novel technique to use cells displaying tANCHORed antigens on the surface for rapid immunization of mice. We showed that transfected HEK293T cells can be injected into mice to induce specific antibodies against the tANCHORed antigen. For efficient induction of antibodies against subunits of a protein, it is very important to choose an expression system that provides a suitable platform to enhance the antigenicity of the subunit protein [41]. In this approach, the tANCHOR system provides an ideal display system to insert small or larger peptides for presenting them on the cell surface. The use of HEK293T cells as a workhorse for the expression of human proteins has several advantages. HEK293T cells are easy to cultivate, they can be transfected with liposomal transfection reagents, they can be detached from the cell culture plate without trypsinization and are capable of expressing high amounts of recombinant proteins [42]. Another advantage is that HEK293T cells are an allogenic material for mice and with an immunization scheme of four injections of whole cells separated by a week, antibodies can be efficiently induced against membrane proteins [43].We suppose that the use of allogenic material triggers immune activation by creating a local immunostimulatory environment. Moreover, the system we have developed includes chimeric fusion of a membrane-bound antigen to increase the antibody response. This same effect has been observed for liposome-connected antigens during immunization experiments [44–47]. These reports showed that presenting an antigen in a membrane environment is beneficial to induce antibodies against a target epitope more efficiently. This observation is supported by the fact that the total amount of antigen that was used for immunization was very low, 1.4 to 1.6 µg for RBD_318–543_, 0.8 µg for RBD_462–510_, and 0.35 µg for cCD20. Conventional doses for immunization contain between 20 and 100 µg of antigen mixed with adjuvant [32, 48–50]. Notably, the best antibody yield was achieved when the BALB/c mice were immunized with the construct displaying the cCD20 loop on the surface. This can be explained by the higher protein expression of the tANCHORed construct bearing cCD20 because of the length of the inserted sequence. Another reason is that the transfection efficiency of the cells transfected with three variants RBD_318–543_ was not quite high enough. Indeed, the transfection efficiency varied between 46.2% and 69.9%. For the RBD_318–543_ of Wuhan and Delta variants, we induced RBD variant-specific antibodies. However, for the Omicron RBD_318–543_, the induction was three times lower than for the Wuhan variant when tested on the spike S1 protein (Fig. 5A–C). In addition, induced antibodies against the Omicron RBD_318–543_ were not highly reactive against the ancestral S1 protein. Additional mutation sites cause the Omicron variant to escape the majority of existing SARS-CoV-2 epitopes where neutralizing antibodies are bound [51]. Interestingly, the direct comparison using coated ELISA plates with variant-specific RBD proteins revealed that the reactivity against the Omicron RBD by antibodies induced with the Omicron variant is more than two times lower than that observed for the Wuhan or Delta variants (Fig. 4E). Antibodies showed cross-reactivity for the Wuhan and Delta variants and low cross-reactivity for the Omicron RBD. BALB/c mice failed to induce sufficient amounts of anti-Omicron IgGs. The Western blot analysis indicates that the protein expression of the tANCHORed Omicron RBD shows a tendency to lower protein amount compared to the Wuhan and Delta RBDs. Although protein expression quantification of tANCHORed Omicron RBD employing the mCherry reporter protein reached statistical significance when compared to other RBD the protein amount of the Omiron RBD showed also a trend to slightly lower protein expression (Fig. 2C and D). But this slightly lower amount does not necessarily lead to a lower induction of antibodies because the induction of antibodies against the FLAG control epitope is comparable to other RBD variant immunizations (Supplementary Fig. S1 C). More importantly, it has been documented that the SARS-CoV-2 Omicron variant evades the immune system by reduced B-cell antigenicity of the RBD [52]. We suppose that the reported lower antigenicity is the main reason why we were not able to detect IgG against the Omicron variant at the same amount as for the Wuhan or Delta variant. In our immunization study, the Wuhan variant yielded the highest amount of IgG that was able to bind to the ELISA plate coated with RBD-specific protein. Taken together, our immunization approach is able to induce antibodies against an antigen that is displayed by the tANCHOR system. Moreover, the obtained immune serum of mice immunized with the full-length RBDs of Wuhan and Delta contains reliable neutralization activity for blockage of the RBD–ACE2 interaction. Further immunization studies are needed to strategically optimize the tANCHORed-based cell immunization. In the case of optimization, different transfection reagents should be tested, aiming to improve protein expression that could result in an increased yield of specific antibodies. The antigen that is displayed using the tANCHOR system has to be inserted between the 3 and 4 transmembrane domain of CD82 by short linker sequences. Optimized amino acid sequence of the linkers in the membrane proximity can improve displaying the antigen. We previously reported that serine- and arginine-free linkers are particularly preferred to enhance protein localization on the surface [53]. If possible, the antigen should not be connected to a high content of arginine and serine N- and C-terminally. We assume that our technique might be of specific interest to induce antibodies against extracellular domains of G protein-coupled receptors (GPRCs). The extracellular surface topology (Fig. 6A) contains an N-terminal domain and three ECLs [54]. The extracellular domains of GPRCs represent an important target where the majority of epitopes are located to which most functional antibodies have been raised [55, 56]. The tANCHOR system could be utilized as an immunogen to provide natively structured GPCR ECLs to generate functional antibodies. Compared to other approaches using a single spanning membrane domain obtained from the platelet-derived growth factor receptor (PDGFR), as demonstrated for displaying HCV E2 antigen, will require the addition of an N-terminal signal peptide and therefore a loop structured displaying of GPCR antigens is not possible (Fig. 6B) [57, 58]. To induce antibodies against the second ECL (ECL2) of GPCRs, commonly used methods include animal immunization with a synthetic peptide as shown for targeting cholecystokinin-B receptor that is highly relevant to gastric or gastrointestinal cancers [59]. The use of GPCRs subunits is useful to reduce immune response against irrelevant epitopes but screening for functional antibodies that bind to native GPCR loops could be laborious. Peptides are easy to obtain for immunization purposes but they lack a native conformation and relevant post-translational modifications of ECLs [60]. To avoid using peptides for animal immunization the use of whole-cell or membrane fraction immunization is possible as shown for targeting the glucagon receptor (GCGR) or C–C chemokine receptor 5 [61, 62]. Unfortunately, full-length GPCRs are weakly expressed at the cell surface without ectopic expression. This results in a high proportion of non-specific immune responses when whole-cell immunization with endogenously expressed GPCRs is used [55, 63]. A combination of the native presentation of GPCRs in a loop structure environment, the use of GPCR subunits such as ECL and high expression on the cell surface would lead to a major GPCR antigen improvement for immunization (Fig. 6C). Therefore, the presentation of the loop structures provided by the tANCHOR system constitutes the most suitable method for example, for GPCRs immunization studies that should be evaluated in future experiments. The main advantage of employing the tANCHOR system is to present GPCR loops in the same topology as they would be presented by a full-length GPCR protein on the cell surface. Although whole-cell immunization is well-established for full-length proteins [43, 64, 65], we showed that with the tANCHOR display technology, it is possible to induce antibodies against short or longer partial protein sequences that are displayed on the cell surface. This enables specific antigen designs for expression at the cell’s surface with desired native expressed loop structures that are essential for the successful production of therapeutic human anti-GPCR antibodies.

Overall, this study identifies antigens anchored by the tANCHOR system as a key to designing effective antigens in order to induce antibodies against a target that is part of the chimeric CD82-derived membrane-bound protein. Our developed technique provides a fast and reliable method to immunize mice without the need for purified antigens. This can speed up immunization projects where mAbs must be generated or to test the antigenicity of different targets for further vaccine studies.

## Supplementary Material

bpad030_Supplementary_Data

## Data Availability

The data that support the findings of this study are available from the corresponding author upon reasonable request.
