# Supplementary material for: tANCHOR fast and cost-effective cell-based immunization approach with focus on the receptor-binding domain of SARS-CoV-2
Source: Biol Methods Protoc. 2023 Dec 12;8(1):bpad030. doi: 10.1093/biomethods/bpad030 (PMC10713279; doi:10.1093/biomethods/bpad030)
Supplement: bpad030_Supplementary_Data [file bpad030_supplementary_data.docx]

**Supplementary Information**

Manuscript number: BMP-2023-054

**tANCHOR fast and cost-effective cell-based immunization approach with focus on the receptor-binding domain of SARS-CoV-2**

Bernauer, Hubert; Schlör, Anja; Maier, Josef; Bannert, Norbert; Hanack, Katja; Ivanusic, Daniel

**Figure S1:**


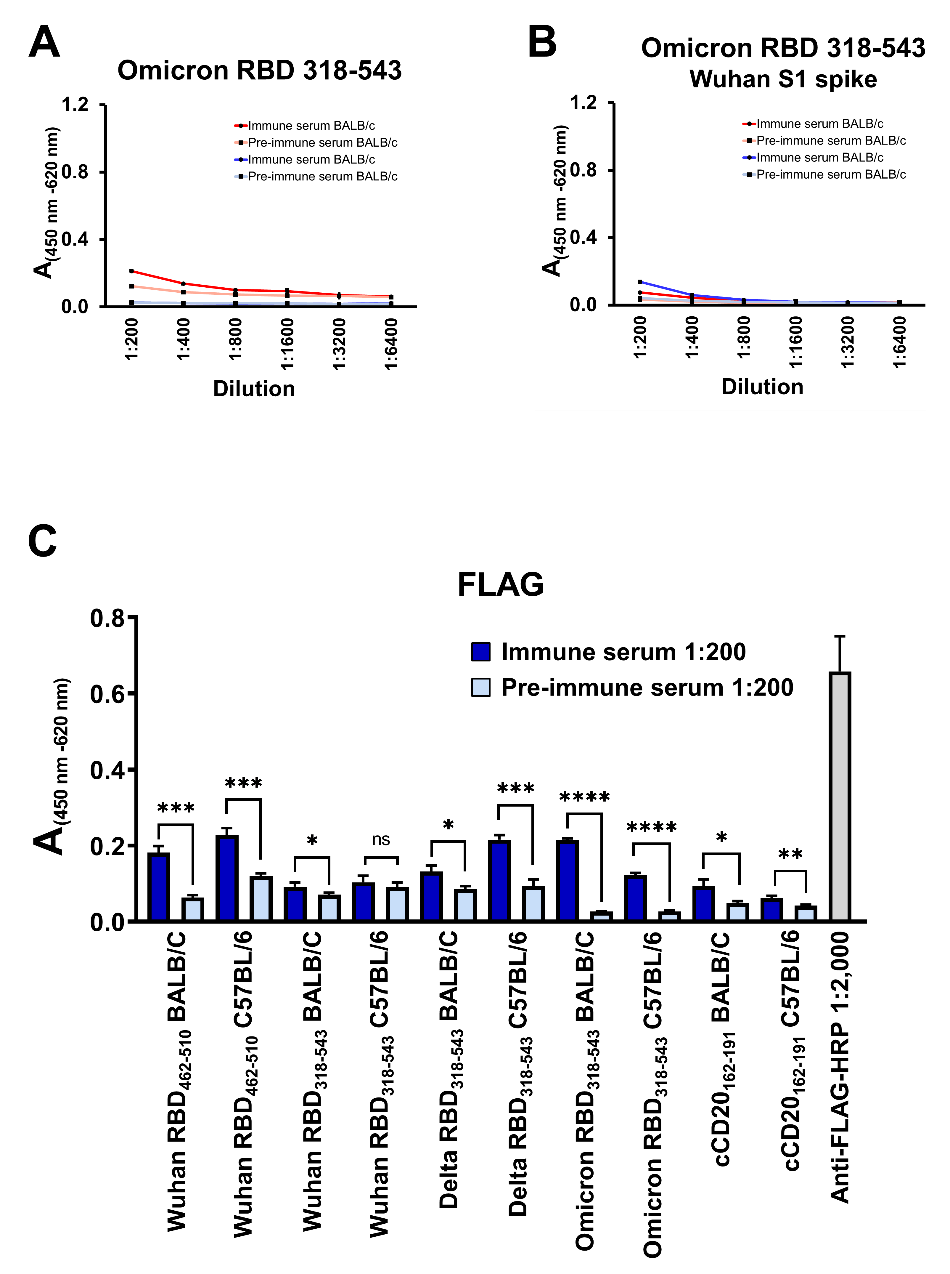


**Supplementary Figure S1:** (**A**) Analysis of IgG induction derived from immunization with tANCHORed Omicron RBD_318-543_ reactive against coated recombinant Omicron RBD and (**B**) against the S1 spike protein. (**C**) Analysis of induced IgG against the N-terminally fused FLAG epitope by a FLAG specific ELISA. The FLAG control was only incubated with an anti-FLAG-HRP antibody. Absorbance values were measured in triplicate, non-significant (ns): > 0.05, *: p < 0.05, **: p < 0.01, ***: p < 0.001, ****: p < 0.0001.

**Supplementary Table 1**

| SARS-CoV-2 Wuhan-Hu-1 RBD gene synthesis fragment GenBank accession: NC_045512.2:22517-23191 |
| --- |
| GAATTCAGAGTCCAACCAACAGAATCTATTGTTAGATTTCCTAATATTACAAACTTGTGCCCTTTTGGTGAAGTTTTTAACGCCACCAGATTTGCATCTGTTTATGCTTGGAACAGGAAGAGAATCAGCAACTGTGTTGCTGATTATTCTGTCCTATATAATTCCGCATCATTTTCCACTTTTAAGTGTTATGGAGTGTCTCCTACTAAATTAAATGATCTCTGCTTTACTAATGTCTATGCAGATTCATTTGTAATTAGAGGTGATGAAGTCAGACAAATCGCTCCAGGGCAAACTGGAAAGATTGCTGATTATAATTATAAATTACCAGATGATTTTACAGGCTGCGTTATAGCTTGGAACTCTAACAATCTTGATTCTAAGGTTGGTGGTAATTATAATTACCTGTATAGATTGTTTAGGAAGTCTAATCTCAAACCTTTTGAGAGAGATATTTCAACTGAAATCTATCAGGCCGGTAGCACACCTTGTAATGGTGTTGAAGGTTTTAATTGTTACTTTCCTTTACAATCATATGGTTTCCAACCCACTAATGGTGTTGGTTACCAACCATACAGAGTAGTAGTACTTTCTTTTGAACTTCTACATGCACCAGCAACTGTTTGTGGACCTAAAAAGTCTACTAATTTGGTTAAAAACAAATGTGTCAATTTCAACTTCGATATC |
| SARS-CoV-2 Wuhan-Hu-1 RBD protein sequence GenBank accession: YP_009724390.1:318-543 |
| EFRVQPTESIVRFPNITNLCPFGEVFNATRFASVYAWNRKRISNCVADYSVLYNSASFSTFKCYGVSPTKLNDLCFTNVYADSFVIRGDEVRQIAPGQTGKIADYNYKLPDDFTGCVIAWNSNNLDSKVGGNYNYLYRLFRKSNLKPFERDISTEIYQAGSTPCNGVEGFNCYFPLQSYGFQPTNGVGYQPYRVVVLSFELLHAPATVCGPKKSTNLVKNKCVNFNFDI |
| Note: Underlined sequence contains the flanked restriction sites *Eco*RI and *Eco*RV. *Eco*RI is coding for EF and therefore F is part of the RBD that is corresponding to amino acid position 318 GenBank: YP_009724390.1. |
| \| **Sequence for construction of Gamma and Omicron RBD variants**  **Data Availability**  GISAID Identifier: EPI_SET_230215my  doi: 10.55876/gis8.230215my  All genome sequences and associated metadata in this dataset are published in GISAID’s EpiCoV database. To view the contributors of each individual sequence with details such as accession number, Virus name, Collection date, Originating Lab and Submitting Lab and the list of Authors, visit [10.55876/gis8.230215my](https://epicov.org/epi3/epi_set/230215my)  **Data Snapshot** EPI_SET_230215my is composed of 9 individual genome sequences.The collection dates range from 2019-12-30 to 2021-11-20; Data were collected in 7 countries and territories; All sequences in this dataset are compared relative to hCoV-19/Wuhan/WIV04/2019 (WIV04), the official reference sequence employed by GISAID (EPI_ISL_402124). Learn more at <https://gisaid.org/WIV04>**.** \| \| \| \| \| \| --- \| --- \| --- \| --- \| --- \| \|  \|  \|  \| **Position in genome** \| \| \| **SARS-CoV-2 variant** \| **GISAID EPI_ISL** \| **Strain** \| **Start** \| **End** \| \| Wuhan-Hu-1 \| EPI_ISL_402124.1 \| hCoV-19/Wuhan/WIV04/2019 \| 22517 \| 23191 \| \| Delta B.1.617.2 \| EPI_ISL_2378732.1 \| hCoV-19/Japan/TKYTK1734/2021 \| 22507 \| 23181 \| \| Omicron BA.1 \| EPI_ISL_6640916.1 \| hCoV-19/Botswana/R40B59_BHP_3321001248/2021 \| 22442 \| 23116 \| \| Omicron BA.1 \| EPI_ISL_6704867.1 \| hCoV-19/South_Africa/NICD-N21668/2021 \| 22493 \| 23167 \| \|  \|  \|  \|  \|  \| \| Note: The RBD coding sequence is 675 base pairs long and encodes 225 amino acids. It represents the amino acids 319-543 of the spike protein (according to Wuhan-1 numbering). For Omicron BA-1, two type strains were respectively considered with the same outcome. The RBD coding sequences of Omicron BA.1 strains are identical to each other, respectively. The *Eco*RI restriction recognition sites at position 354-359 GAATTC, with positions counted from the start of codon F318, present within the RBD-coding sequences in all strains, were removed by silent base changes 357TC. N-terminal *Eco*RI is coding for EF and therefore F is part of the RBD that is corresponding to aa position 318.   \| **Variant** \| **Mutations within the RBD** \| \| --- \| --- \| \| Delta B.1.617.2 \| L452R, T478K \| \| Omicron BA.1 \| G339D, S371L, S373P, S375F, K417N, N440K, G446S, S477N, T478K, E484A, Q493R, G496S, Q498R, N501Y, Y505H \| \| \| \| \| \| |
| **Canine CD20 gene synthesis fragment** |
| GAATTCGTGGACATCCATAATTGTGACCCCGCAAATCCGAGCGAGAAGAACAGCCTGAGTATCCAGTACTGTGGCAGCATCAGAAGTGTGTTTTTGGATATC |
| Note: The underlined sequence contains the flanked restriction sites *Eco*RI and *Eco*RV |
| **Canine CD20 translated sequence Genbank: NP_001041493.1:162-191** |
| EFVDIHNCDPANPSEKNSLSIQYCGSIRSVFLDI |
| **Human angiotensin-converting enzyme 2 (ACE2) gene synthesis fragment**  Sequence was fused with coding sequence for V5 and 6 x His tag |
| gctagcaatttataacgaga**atgagcagcagcagctggctgctgctgagcctggtggccgtgaccgccgcccagagcaccatcgaggagcaggccaagaccttcctggacaagttcaaccacgaggccgaggacctgttctaccagagcagcctggccagctggaactacaacaccaacatcaccgaggagaacgtgcagaacatgaacaacgccggcgacaagtggagcgccttcctgaaggagcagagcaccctggcccagatgtaccccctgcaagagatccagaacctgaccgtgaagctgcaactgcaagccctgcaacagaacggcagcagcgtgctgagcgaggacaagagcaagcgcctgaacaccatcctgaacaccatgagcaccatctacagcaccggcaaggtgtgcaaccccgacaacccccaggagtgcctgctgctggagcccggcctgaacgagatcatggccaacagcctggactacaacgagcgcctgtgggcctgggagagctggcgcagcgaggtgggcaagcagctgcgccccctgtacgaggagtacgtggtgctgaagaacgagatggcccgcgccaaccactacgaggactacggcgactactggcgcggcgactacgaggtgaacggcgtggacggctacgactacagcaggggccagctgatcgaggacgtggagcacaccttcgaggagatcaagcccctgtacgagcacctgcacgcctacgtgcgcgccaagctgatgaacgcctaccccagctacatcagccccatcgggtgcctgcccgcccacctgctgggcgacatgtggggccgcttctggaccaacctgtacagcctgaccgtgcccttcggccagaagcccaacatcgacgtgaccgacgcgatggtggaccaggcctgggacgcccagcgcatcttcaaggaggccgagaagttcttcgtgagcgtgggcctgcccaacatgacccagggcttctgggagaacagcatgctgaccgaccccggcaacgtgcagaaggccgtgtgccaccccaccgcctgggacctgggcaagggcgacttccgcatcctgatgtgcaccaaggtgactatggacgacttcctgaccgcccaccacgagatgggccacatccagtacgacatggcctacgccgcccagcccttcctgctgcgcaacggcgccaacgagggcttccacgaggccgtgggcgagatcatgagcctgagcgccgccacccccaagcacctgaagagcatcggcctgctgagccccgacttccaggaggacaacgagaccgagatcaacttcctgctgaagcaggccctgaccatcgtgggcaccctgcccttcacctacatgctggagaagtggcgctggatggtgttcaagggcgagatccccaaggaccagtggatgaagaagtggtgggagatgaagcgcgagatcgtgggcgtggtggagcccgtgccccacgacgagacctactgcgaccccgccagcctgttccacgtgagcaacgactacagcttcatccgctactacacccgcaccctgtaccagttccagttccaggaggccctgtgccaggccgccaagcacgagggtcccctgcacaagtgcgacatcagcaacagcaccgaggccggccagaagctgttcaacatgctgcgcctgggcaagagcgagccctggaccctggccctggagaacgtggtgggcgccaagaacatgaacgtgcgccccctgctgaactacttcgagcccctgttcacctggctgaaggaccagaacaagaacagcttcgtgggctggagcaccgactggagcccctacgccgaccagagcatcaaggtgcgcatcagcctgaagagcgccctgggcgacaaggcctacgagtggaacgacaacgagatgtacctgttccgcagcagcgtggcctacgccatgcgccagtacttcctgaaggtgaagaaccagatgatcctgttcggcgaggaggacgtgcgcgtggccaacctgaagccccgcatcagcttcaacttcttcgtgaccgcccccaagaacgtgagcgacatcatcccccgcaccgaggtggagaaggccatccgcatgagccgcagccgcatcaacgacgccttccgcctgaacgacaacagcctggagttcctgggcatccagcccaccctgggtccccccaaccagccccccgtgagc**ggaaagggcccgcggttcgaaggtaagcctatccctaaccctctcctcggtctcgattctacgcgtaccggtcatcatcaccatcaccattgagtttaaac |
| Note: DNA sequence coding for ACE2 amino acids 1-740 is bold displayed, sequence coding for the V5-6xHis tag is underlined, other sequence 5´and 3´ is used for cloning in the target vector using the restriction sites *Nhe*I/*Pme*I. |
| Angiotensin-converting enzyme 2 isoform 2 precursor [Homo sapiens]  GenBank RefSeq Protein: NP_001373188.1:1-740 |
| **MSSSSWLLLSLVAVTAAQSTIEEQAKTFLDKFNHEAEDLFYQSSLASWNYNTNITEENVQNMNNAGDKWSAFLKEQSTLAQMYPLQEIQNLTVKLQLQALQQNGSSVLSEDKSKRLNTILNTMSTIYSTGKVCNPDNPQECLLLEPGLNEIMANSLDYNERLWAWESWRSEVGKQLRPLYEEYVVLKNEMARANHYEDYGDYWRGDYEVNGVDGYDYSRGQLIEDVEHTFEEIKPLYEHLHAYVRAKLMNAYPSYISPIGCLPAHLLGDMWGRFWTNLYSLTVPFGQKPNIDVTDAMVDQAWDAQRIFKEAEKFFVSVGLPNMTQGFWENSMLTDPGNVQKAVCHPTAWDLGKGDFRILMCTKVTMDDFLTAHHEMGHIQYDMAYAAQPFLLRNGANEGFHEAVGEIMSLSAATPKHLKSIGLLSPDFQEDNETEINFLLKQALTIVGTLPFTYMLEKWRWMVFKGEIPKDQWMKKWWEMKREIVGVVEPVPHDETYCDPASLFHVSNDYSFIRYYTRTLYQFQFQEALCQAAKHEGPLHKCDISNSTEAGQKLFNMLRLGKSEPWTLALENVVGAKNMNVRPLLNYFEPLFTWLKDQNKNSFVGWSTDWSPYADQSIKVRISLKSALGDKAYEWNDNEMYLFRSSVAYAMRQYFLKVKNQMILFGEEDVRVANLKPRISFNFFVTAPKNVSDIIPRTEVEKAIRMSRSRINDAFRLNDNSLEFLGIQPTLGPPNQPPVS**GKGPRFEGKPIPNPLLGLDSTRTGHHHHHH |
| Note: Bold displayed protein sequence contains the amino acids of the ACE2 1-740 followed by the fused tags V5 and 6 x His that is underlined. |
